# Case Reports: Presumable tuberculous mastitis-like

**DOI:** 10.1590/0037-8682-0344-2023

**Published:** 2024-09-02

**Authors:** Michel de Araujo Tavares, Marcelo Cordeiro dos Santos, Vinicius da Silva Monteiro, Djane Baia-da-Silva, Marcus Vinícius Guimarães de Lacerda, René Aloisio da Costa Vieira

**Affiliations:** 1 Universidade Estadual Paulista “Júlio de Mesquita Filho”, Faculdade de Medicina de Botucatu, Programa de Pós-Graduação em Tocoginecologia, Botucatu, SP, Brasil.; 2 Universidade Federal do Amazonas, Faculdade de Medicina, Departamento de Clínica Médica, Manaus, AM, Brasil.; 3 Fundação de Medicina Tropical Doutor Heitor Vieira Dourado, Manaus, AM, Brasil.; 4 Universidade do Estado do Amazonas, Programa de Pós-Graduação em Medicina Tropical, Manaus, AM, Brasil.; 5 Fundação de Medicina Tropical Doutor Heitor Vieira Dourado, Serviço de Assistência Especializada, Manaus, AM, Brasil.; 6 Universidade Nilton Lins, Manaus, AM, Brasil.; 7 Fundação Oswaldo Cruz/Instituto Leônidas & Maria Deane, Instituto de Pesquisa Clínica Carlos Borborema, Manaus, AM, Brasil.

**Keywords:** Granulomatous mastitis, Extrapulmonary tuberculosis, Presumptive tuberculous mastitis

## Abstract

Tuberculous mastitis (TM) is a rare cause of breast disease. The test sensitivity for TM is low and sometimes characterized by idiopathic granulomatous mastitis. Both have different treatments; however, many patients are treated with presumptive TM (P-TM). We present fifteen women with confirmed TM or P-TM. We found 4% (5/124) of TM and 8% (10/124) of P-TM, grouped according to their diagnosis, with no differences between them, except for follow-up and treatment time. P-TM is an entity that must be considered based on clinical and radiological suspicion and is associated with presumptive pathological data.

## INTRODUCTION

One third of the world's population has had contact with *Mycobacterium tuberculosis*, which causes tuberculosis (TB). TB is more common in developing countries, with 22 countries accounting for 80% of the affected individuals; Brazil ranks 16th in incidence. Tuberculous mastitis (TM) is a rare, but an important cause of breast infections that are difficult to diagnose^1^. The incidence of TM ranges from 0.1% in developed countries to approximately 4% in highly TB endemic countries^2^. In the Amazon, where TB is endemic, there has been only one TM report^3^. Patients with mastitis are usually treated with antibiotic therapy, and among those without resolution, TM should be differential. The sensitivity of tests for the diagnosis of TM is low (approximately 12% for *M. tuberculosis* culture, the gold standard technique), and the histological diagnosis is idiopathic granulomatous mastitis (IGM)^4^ ^,^ ^5^. Both have different treatments; however, many patients are treated with presumptive TM (P-TM). We present the clinical aspects and outcomes of 15 women with confirmed TM or P-TM-treated who showed a clinical response at Fundação de Medicina Tropical Doutor Heitor Vieira Dourado, a reference service for infectious diseases in the Brazilian Amazon, located in Manaus, Amazonas, between 2013 and 2021. We defined antituberculosis therapeutic response as a diagnostic criterion.

Clinical, laboratory, and radiological data were obtained from the electronic medical records (iDoctor system). For the etiological diagnosis of TM, we consider the results of multiple procedures: fine needle aspiration; core needle biopsy (CNB); and laboratory tests, such as the purified protein derivative (PPD) skin test, Ziehl-Neelsen (ZN) staining, culturing, polymerase chain reaction, Xpert MTB RIF rapid molecular test (RMT), bacilloscopy (acid fast bacilli -AFB BAAR), histopathological evaluation, culture for identification of mycobacterium and aerobic bacteria, direct examination, and culture of fungi. For P-TM, the following were considered: (1) clinical mastitis, which is difficult to resolve with usual therapies; (2) PPD skin test positivity; (3) histology suggestive of IGM or nonspecific chronic mastitis; and (4) absence of recurrence during a minimum follow-up period of six months after treatment.

## CASE REPORT

**FIGURE 1:**
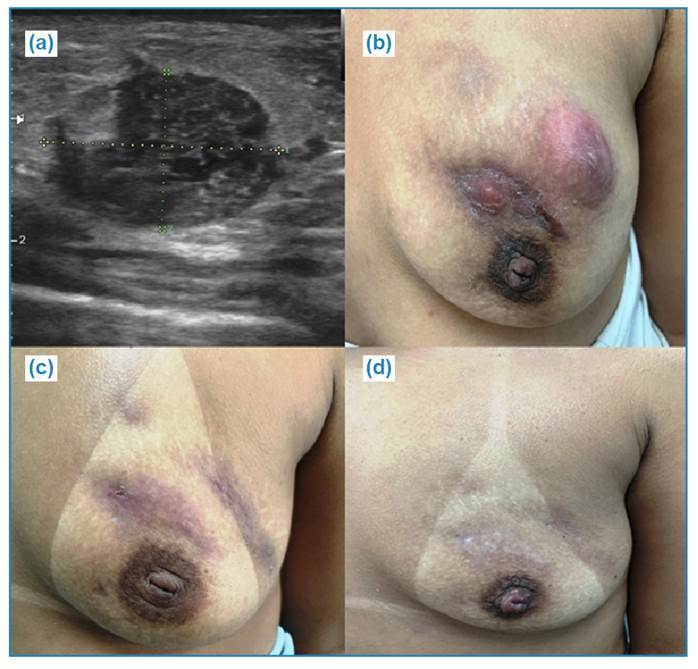
Patient with a presumed diagnosis of breast tuberculosis. **a)** Breast ultrasound showing a solid hypoechoic nodule with circumscribed margins in the left breast. **b)** Left breast showing abscesses, scars, and fistulas in the sixth week of treatment for breast tuberculosis. **c)** Left breast showing abscesses, scars, and fistulas on the third month of treatment for breast tuberculosis. **d)** Left breast showing clinical improvement 10 months after starting treatment.

**FIGURE 2:**
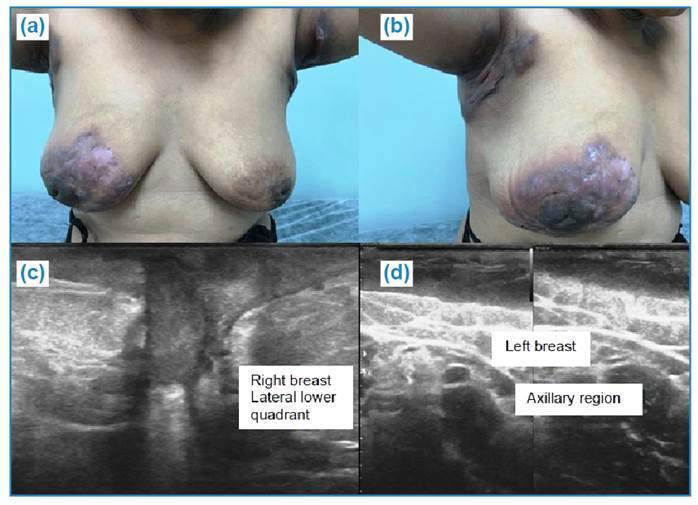
Patients in the third month of treatment for bilateral mammary and lymph node tuberculosis. **a)** and **b)** Images of the breast and axillary regions with scars, fistulas, erythema, and skin thickening. **c)** Breast ultrasonography demonstrating a fistulous path with a collection of thick fluid intermingled in the inferolateral quadrant of the right breast. **d)** Breast ultrasound showing skin thickening in the left axillary region.

One hundred and twenty-four patients with IGM were identified throughout the study period: 5 and 10 were diagnosed with TM and P-TM, respectively. The clinical, laboratory, and radiological characteristics of the evaluated women are shown in Table 1. The average age among those diagnosed with TM was 35.4 (range: 22-50), while the average age among those with P-TM was 35.1 (range: 24-62). None of the patients were diagnosed with HIV; however, patients 1, 2, and 3 previously had TB. Most patients complained of pain, heat, breast erythema, breast lumps, and fistulous disease. Fever, chills, and axillary adenopathy were the most common systemic complaints. All patients had positive PPD (induration equal to or greater than 5 mm). Among the five patients with TM, only one had a bacteriologically confirmed positive culture. Altered mammography findings were nonspecific, and the ultrasound findings were highlighted. The ultrasonographic findings were more expressive. Six patients had abscesses and six had fistulas. The most common findings were fistulas, abscesses, circumscribed nodules, hypoechoic nodules, and skin thickening (Figures 1 and 2). Magnetic resonance imaging and chest computed tomography were performed for only one patient and no specific findings were observed. We grouped the individuals according to their diagnosis of TM and P-TM and found no differences between the groups, except for the follow-up and treatment time, which were shorter in the TM group, as expected (Supplementary Table 1), given the need for minimal follow-up and no recurrence.

**TABLE 1:** Clinical, laboratory, and radiological aspects per case.

ID	Mastitis classification	Age	Local symptoms	Systemic symptoms	Radiological aspects	Histology	Criteria	PPD (induration in mm)	Time of treatment (months)	Time of follow-up time (months)	Ultrasound
1	TM	22	Pain, erythema, papillary discharge, nodule/mass, and fistula	Fever and chills, axillary lymph nodes	Abscess and fistula	NP	AFB positive in respiratory sample	NP	6.2	8.08	Sclerosant
2	TM	41	Pain, erythema, papillary discharge, and nodule/mass	Fever and chills	Circumscribed hypoechoic nodule	ICM (ZN+)	AFB positive in respiratory sample	NP	8.8	1.66	Nodular
3	TM	24	Pain, erythema, papillary discharge, nodule/mass, and fistula	Axillary adenopathy	Abscess and fistula	GCMc	PPD, lung TB and GCM	13	6.0	4.39	Nodular
4	TM	50	Pain, erythema, papillary discharge, and abscess	Fever and chills	Hypoechoic nodule	GCMc (ZN+)	PPD, Biopsy	8	6.0	0.01	Nodular
5	TM	40	Pain, erythema, papillary discharge, nodule/mass, and abscess	Axillary adenopathy	Circumscribed hypoechoic nodule	ICM	Positive culture	NP	9.0	3,89	NP
6	P-TM	39	Erythema, papillary discharge, and nodule/mass	Fever and chills, axillary adenopathy	Abscess, irregular hypoechoic nodule, architectural distortion	GCM	PPD and GCM	25	8.0	24.17	Nodular
7	P-TM	62	Pain, erythema, nodule/mass, and abscess	Axillary adenopathy	Fistula and skin thickening	ICM	PPD	21	9.3	9.91	Diffuse
8	P-TM	26	Pain, erythema, nodule/mass, and abscess	Fever and chills	Circumscribed hypoechoic nodule and axillary adenopathy.	GCM	PPD and GCM	12	6.0	6.66	Nodular
9	P-TM	41	Pain, erythema, nodule/mass, and fistula	Fever and chills	Fistula	GCMc	PPD, GCM, IGRA	25	6.0	14.1	Sclerosant
10	P-TM	29	Pain, erythema, nodule/mass, and fistula	-	Abscess	GCM	PPD and GCM	14	9.0	15.11	Nodular
11	P-TM	34	Pain, erythema nodule/mass, and fistula	Fever and chills, axillary adenopathy	Confluent nodules	ICM	PPD	19	9.1	18.47	Nodular
12	P-TM	33	Pain, erythema, and papillary discharge		Abscess and axillary adenopathy	ICM	PPD	18	8.3	6.01	Diffuse
13	P-TM	24	Pain, erythema, papillary discharge, abscess and fistula	Fever and chills	Abscess and axillary adenopathy	ICM	PPD	17	9.0	6.03	Sclerosant
14	P-TM	29	Pain, erythema, papillary discharge, abscess and fistula	-	Fistula	ICM	PPD and CT	15	10.1	8.78	Diffuse
15	P-TM	34	Pain, erythema, and fistula	-	Fistula	GCM	PPD and GCM	5	7.1	26.06	Nodular

**AFB:** acid-fast bacilli; **IGRA:** interferon gamma release assay; **TM:** tuberculous mastitis; **P-TM:** presumable-tuberculous mastitis; **GCMc:** caseous granulomatous chronic mastitis; **GCM:** granulomatous chronic mastitis; **ICM:** non-specific chronic mastitis; **CT:** computed tomography; **NP:** not performed; **PPD:** purified protein derivative; **ZN:** Ziehl-Neelsen stain; **TB:** Tuberculosis. Lung TB was diagnosed by lung biopsy.

## DISCUSSION

TB is common in underdeveloped and developing countries, such as Africa, India, and Pakistan^4^. In Brazil, TB is endemic to the Amazon, with an estimate is 74.1 per 100,000 inhabitants. In Manaus, the rate is 93.2 per 100,000 inhabitants and is associated with income inequalities that influence health and TB transmission^6^. Although TB is a common disease in the Amazon region, we found 4% TM and 8% P-TM cases in women treated with idiopathic granulomatous mastitis at a reference hospital for infectious diseases in the Brazilian Amazon. The low frequency of TM can be explained by the diagnostic difficulty, which is generally defined as IGM. The importance of the diagnostic definition is that the treatment differs, since IGM is usually treated with surgery or immunosuppressive drugs, such as corticosteroids and methotrexate, which are harmful if the patient has TM. In addition, it is harmful to the patient undergoing a surgical procedure when there is a treatable etiology of a disease that may be recurrent; therefore, the etiology must be extensively evaluated. However, IGM is treated with immunosuppressants and has a high recurrence rate^7^. One treatment option for IGM is surgery, including mastectomy. The problem is performing surgery when there is a treatable etiology; therefore, the etiology must be evaluated extensively.

In the present study, the diagnostic method varied according to the medical criteria. However, we adjusted the definitions and re-evaluated the classification of cases. According to medical records, 23 women had TM or P-TM; however, based on a detailed analysis of the kinetics, diagnostic methods used, clinical characteristics, and response to therapy, we found only 15 cases. Diagnostic evaluation is essential, and many of these patients are treated with antibiotic therapy and have persistent or recurrent breast findings^4^. Patients should be evaluated histologically in the absence of resolution. In the presence of granulomatous lesions, IGM, TM should be considered as differential diagnosis^8^. The gold standard for the diagnosis of the disease is the detection of the etiologic agent, *M. tuberculosis* using Ziehl-Neelsen staining or culture. However, AFB smear positivity and ZN staining are not frequently performed. In most cases, breast TB can only be accurately diagnosed using the histological identification of a typical necrotizing granulomatous lesion^9^. On biopsy, the predominance of neutrophils in the background and relative absence of caseous necrosis favor the diagnosis of granulomatous mastitis. Generally, CNB is sufficient for evaluation, therefore, an open biopsy is rarely necessary^10^. In TB-endemic areas, the PPD skin test may be positive and related to previous exposure to *M. tuberculosis* in those vaccinated with the Bacillus Calmette-Guerin vaccine or previous exposure to nontuberculous mycobacteria. It is difficult to distinguish between an old and recent infection; therefore, the PPD skin test lacks diagnostic value^11^. In our study, almost all patients treated for TM showed elevated skin test results. 

The Brazilian study included 20 cases, of which 19 women had IGM with caseous necrosis and the ZN test was positive in only one patient. All the patients had high PPD levels. The patients were treated for six months, with a mean follow-up of 12 months (8-14 months), and no recurrence was observed^12^. Despite having 15 patients (five with TM and 10 with P-TM), our study constitutes one of the largest Brazilian studies influenced by the endemic area and treatment in specific treatment centers. PPD values and follow-up times were similar.

This study had some limitations, particularly those associated with the study design. However, it represents the second largest sample in Brazil and originates from an endemic region. The lack of positivity criteria for all patients was overcome by the response to therapy and the six-month follow-up of patients with P-TM. This study aimed to establish the criteria based on the presence of a therapeutic response. 

In conclusion, standardizing diagnostic tests can increase the positivity rate for TM. P-TM must be considered based on clinical and radiological suspicion, associating presumptive pathological data with examinations that indirectly suggest exposure to TB. Among the treated patients, the anti-TB therapeutic response must be considered, and one of the criteria for the final diagnosis is the response to long-term therapy.
