# Supplementary material for: Case Reports: Presumable tuberculous mastitis-like
Source: Rev Soc Bras Med Trop. 2024 Sep 2;57:e00806-2024. doi: 10.1590/0037-8682-0344-2023 (PMC11290841; doi:10.1590/0037-8682-0344-2023)
Supplement: Supplementary file 1 [file 1678-9849-rsbmt-57-e00806-2024-supp1.pdf]

SUPPLEMENTARY TABLE 1: Clinical, diagnostic, and follow-up characteristics of women with TM and P-TM.

Variable	TM (n=5)	P-TM (n=10)	Total (n=15)	P**
**Age** *	35.4 ±12.0 (22, 50)	35.1 ± 10.9 (24, 62)	35.2 (22, 62)	0.95
**Local symptoms**				
Pain	5	9	14	1.00
Heat	5	10	15	-
Erythema	5	10	15	-
Abscess closed	2	5	7	1.00
Abscess fistulous	2	6	8	0.61
Nodule	4	6	10	0.60
Fistula and retraction	0	4	4	0.23
**Systemic symptoms**				
Fever	3	5	8	1.00
Chills	0	3	3	0.50
Weight loss	2	3	5	1.00
Axillary adenopathy	3	3	6	0.33
Cough	0	2	2	0.52
**Risk factors**				
Previous TB	3	0	3	0.03
Generic mastitis	3	10	13	0.09
Diagnostic Delay	4	10	14	0.33
**PPD***	10.5±3.5 (8–13)	17±6.1 (5–25)	16±6.1 (5–25)	0.18
**Treatment time***	5.2±1.1(4.4–6)	8.1±1.5 (5.7–10)	7.7±1.7(4.4–10)	0.21
**Follow-up***	2.2±3.1(0.1–4.4)	13.4±7.6 (6–26)	10.1±7.9 (0.1–26)	0.01
**Radiological variables of breast ultrasound**				
Irregular hypoechoic nodule	0	1	1	1.00
Axillary adenopathy	0	2	2	0.52
Circumscrite hypoechoic nodule	3	1	4	0.08
Ductal dilatation	0	1	1	1.00
Skin thickening	0	3	3	0.50
Abscess	2	4	6	1.00
Fistula	2	4	6	1.00
Distortion	0	1	1	1.00

TB: tuberculosis; **PPD:** purified protein derivative; **P-TM:** presumably tuberculous mastitis, **TM:** tuberculous mastitis; **SD:** Standard Deviation.

* Presented as average±SD (min–max). ** chi-square test was used, and when a variable had a number lower than 5, Fisher`s exact test was used. When comparing continuous variables using the Mann––Whitney U test.
